# Supplementary material for: Identification of photoperiod-induced specific miRNAs in the adrenal glands of Sunite sheep (Ovis aries)
Source: Front Vet Sci. 2022 Jul 22;9:888207. doi: 10.3389/fvets.2022.888207 (PMC9354845; doi:10.3389/fvets.2022.888207)
Supplement: Supplementary file 1 [file Data_Sheet_1.ZIP › Supplementary file/miRDeep2 file.zip!/miRDeep2 file/Know_Structure/SPLP42A2/oar-mir-19b.pdf]

miRBase precursor : oar-mir-19b
Total read count : 2265
oar-miR-19b read count : 2260
remaining reads : 5

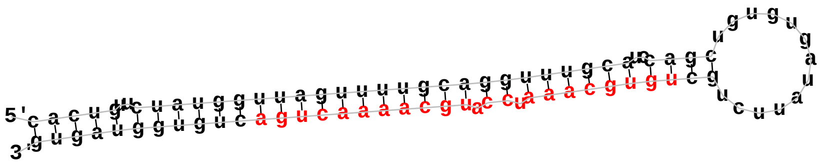

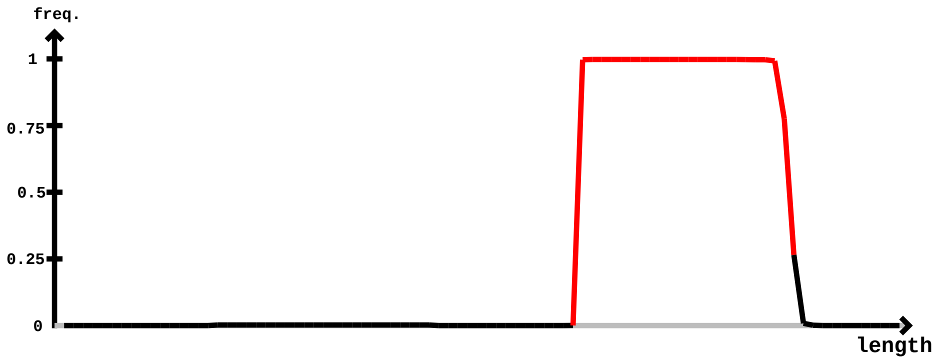

oar-miR-19b

5'- cacuguucuaugguuaguuuugcagguuugcauccagcugugugauauucugcugugcaaauccaugcaaaacugacugugguagug -3'	exp		
(((((..(((((((((((((((((((((((((..((((.............)))))))))).)).))))))))))))))))))))))	reads	mm	sample
...............aguuuugcagguuugcauccagc...	5	0	seq
....cugugcaaauccaugcaaaacu.............	1	0	seq
....ugugcaaauccaugcaa.................	1	0	seq
....ugugcaaauccaugUaaa................	1	1	seq
....ugugcaaauccaugcaaaac..............	9	0	seq
....ugugcaaaucUaugcaaaac..............	1	1	seq
....ugugcaaaucUaugcaaaacu.............	61	1	seq
....ugugcaaauccaugcaaaacu.............	431	0	seq
....ugugcaaauccaugcaaaacug............	1066	0	seq
....ugugcaaaucUaugcaaaacug............	89	1	seq
....ugugcaaauccaugcaaaacuga...........	485	0	seq
....ugugcaaaucUaugcaaaacuga...........	94	1	seq
....ugugcaaauccaugcaaaacugaU..........	15	1	seq
....ugugcaaauccaugcaaaacugac..........	1	0	seq
....ugugcaaauccaugcaaaacugaUu.........	3	1	seq
....gugcaaauccaugcaaaacuga...........	2	0	seq
